# fmcmc: A friendly MCMC framework

**DOI:** 10.21105/joss.01427

**Published:** 2019-07-09

**Authors:** George G Vega Yon, Paul Marjoram

**Affiliations:** 1 Department of Preventive Medicine, University of Southern California

## Summary

Markov Chain Monte Carlo (MCMC) is used in a variety of statistical and computational venues such as: statistical inference, Markov quadrature (also known as Monte Carlo integration), stochastic optimization, among others. The **fmcmc** R (R Core Team, 2019) package provides a flexible framework for implementing MCMC methods that use the Metropolis-Hastings algorithm (Hastings, 1970; Metropolis, Rosenbluth, Rosenbluth, Teller, & Teller, 1953). **fmcmc** provides the following out-of-the-box features that can be valuable for both practitioners of MCMC and educators:

- Seamless efficient multiple-chain sampling using parallel computing,
- User-defined transition kernels, and
- Automatic stop using convergence monitoring.

In the case of transition kernels, users can either use one of the transition kernels shipped with the package (e.g. the Gaussian kernel and its bounded version, the Gaussian kernel with reflective boundaries), this allows a degree of flexibility that is not possible with existing MCMC packages.

The main function automatically checks convergence during execution, and stop the MCMC run once convergence has been reached, rather than having to pre-determine a fixed number of iterations. Users can either use one of the convergence monitoring checking functions that are part of the package, for example: The Gelman and Rubin’s (Gelman, Rubin, & others, 1992), Geweke’s (Geweke & others, 1991), etc. Or build their own to be used within the framework.

While there are several other R packages that either implement MCMC algorithms or provide wrappers for implementations in other languages (see for example: Sturtz, Ligges, & Gelman, 2005; Carpenter et al., 2017; Geyer & Johnson, 2019; Martin, Quinn, & Park, 2011; Morey, 2009; Scheidegger, 2018; Stan Development Team, 2018), to our knowledge, the fmcmc package is the first one to provide a framework as flexible as the one described here and to be implemented fully within R.
